# A phase I, first-in-human study of TAK-164, an antibody–drug conjugate, in patients with advanced gastrointestinal cancers expressing guanylyl cyclase C

**DOI:** 10.1007/s00280-023-04507-w

**Published:** 2023-02-04

**Authors:** Richard Kim, Alexis D. Leal, Aparna Parikh, David P. Ryan, Shining Wang, Brittany Bahamon, Neeraj Gupta, Aaron Moss, Joanna Pye, Harry Miao, Haig Inguilizian, James M. Cleary

**Affiliations:** 1 Department of Gastroenterology Oncology, Moffitt Cancer Center, Vincent A. Stabile Research Building, 12902 USF Magnolia Drive, Tampa, FL 33612 USA; 2 Division of Medical Oncology, University of Colorado School of Medicine, Aurora, CO USA; 3 Division of Hematology and Oncology, Massachusetts General Hospital Cancer Center, Harvard Medical School, Boston, MA USA; 4 Oncology Clinical Science, Takeda Development Center Americas, Inc. (TDCA), Lexington, MA USA; 5 Translational Medicine, Takeda Development Center Americas, Inc. (TDCA), Lexington, MA USA; 6 Quantitative Clinical Pharmacology, Takeda Development Center Americas, Inc. (TDCA), Lexington, MA USA; 7 Pharmacology/Toxicology, Audentes Therapeutics, Inc., San Francisco, CA USA; 8 Oncology Statistics, Takeda Development Center Americas, Inc. (TDCA), Lexington, MA USA; 9 Clinical Development, Takeda Development Center Americas, Inc. (TDCA), Lexington, MA USA; 10 Global Patient Safety and Evaluation, Takeda Development Center Americas, Inc. (TDCA), Lexington, MA USA; 11 Department of Medical Oncology, Dana-Farber Cancer Institute, Harvard Medical School, Boston, MA USA

**Keywords:** Gastrointestinal cancers, Biological agents and therapies, Drug targets, Clinical-stage research, Clinical trial results

## Abstract

**Purpose:**

Guanylyl cyclase C (GCC) is highly expressed in several gastrointestinal malignancies and preclinical studies suggest that it is a promising target for antibody-based therapeutics. This phase I trial assessed the safety and tolerability of TAK-164, an investigational, anti-GCC antibody–drug conjugate (NCT03449030).

**Methods:**

Thirty-one patients with GCC-positive, advanced gastrointestinal cancers received intravenous TAK-164 on day 1 of 21-day cycles. Dose escalation proceeded based on cycle 1 safety data via a Bayesian model.

**Results:**

Median age was 58 years (range 32–72), 25 patients (80.6%) had colorectal carcinoma, and median number of prior therapies was four. No dose-limiting toxicities (DLTs) were reported during cycle 1 DLT evaluation period. After cycle 2 dosing, 3 patients reported dose-limiting treatment-emergent adverse events (TEAEs): grade 3 pyrexia and grade 5 hepatic failure (0.19 mg/kg), grade 4 hepatic failure and platelet count decreased (0.25 mg/kg), grade 3 nausea, grade 4 platelet and neutrophil count decreased (0.25 mg/kg). The recommended phase II dose (RP2D) was 0.064 mg/kg. Common TAK-164-related TEAEs included platelet count decreased (58.1%), fatigue (38.7%), and anemia (32.3%). There was a dose-dependent increase in TAK-164 exposure over the range, 0.032–0.25 mg/kg. TAK-164 half-life ranged from 63.5 to 159 h. One patient (0.008 mg/kg) with high baseline GCC expression had an unconfirmed partial response.

**Conclusions:**

TAK-164 appeared to have a manageable safety profile at 0.064 mg/kg. Hepatic toxicity was identified as a potential risk. The RP2D of 0.064 mg/kg was considered insufficient to derive clinical benefit; there are no plans for further clinical development.

**Clinical Trial Registration:**

NCT03449030.

**Supplementary Information:**

The online version contains supplementary material available at 10.1007/s00280-023-04507-w.

## Introduction

TAK-164 is a novel second-generation anti-guanylyl cyclase C (GCC) antibody–drug conjugate (ADC), consisting of a human immunoglobulin (IgG)1 monoclonal antibody (mAb) specifically targeting GCC, linked to a novel class of DNA alkylating agents termed mono-indolinobenzodiazepine pseudodimers (IGNs), by a peptide linker [1]. GCC is a transmembrane cell surface receptor that is expressed throughout the gastrointestinal (GI) tract and is involved in mucosal homeostasis, genomic integrity, and cell proliferation [2–6]. GCC has been shown to be expressed in various GI cancers, including 98% of colorectal, 68% of stomach, 64% of pancreatic, and 59% of esophageal cancers; furthermore, GCC expression was found to be higher in colorectal tumors than in matched healthy tissues [7–9], and GCC-positive status was maintained throughout tumor progression based on results from immunohistochemistry (IHC) assays of matched colorectal cancer and liver metastasis cases [7]. In a murine model, GCC ligands have been shown to selectively target colon cancer cells without accumulating in healthy intestinal tissue [10]. In healthy intestinal tissue, GCC is apically expressed and, therefore, restricted to the luminal side of the mucosa [11]; however, in primary and metastatic tumor cells, GCC is expressed both apically and in the cytoplasm [7], with higher expression at the apical membrane compared with normal tissue [9]. This distinction results in GCC-expressing cancer cells being accessible through the vascular compartment by the intravenous administration of therapeutic agents [12]. Taken together, these data suggest that GCC is an attractive target for antibody-based therapeutics in GI cancers.

TAK-264, a first-generation anti-GCC ADC consisting of a mAb linked to a microtubule inhibitor, monomethyl auristatin, demonstrated preclinical highly specific cytotoxic activity [12]. In two subsequent phase I studies, TAK-264 had a manageable safety profile and showed preliminary efficacy in patients with a range of GI malignancies [13, 14]. Due to limited efficacy (overall response rate [ORR] was 6%) in a phase II study; however, further investigation of the drug was not warranted [15]. TAK-164 was developed using the same highly specific GCC targeting mAb but utilizing an alternative payload and mode of action to TAK-264 [1]. Once internalized into GCC-expressing tumor cells, TAK-164 subsequently releases a cytotoxic payload, pertaining an IGN [1, 16]. In a preclinical study, TAK-164 was shown to selectively bind to, be internalized by, and exert cytotoxic activity in GCC-expressing cells, including those refractory to TAK-264; in addition, TAK-164 administration resulted in dose-dependent, tumor growth rate inhibition in PHTX models of metastatic colorectal cancer, and TAK-164 uptake was visualized by positron emission tomography and correlated with GCC expression [1]. Based on these encouraging preclinical results, this first in-human, phase I study was designed to evaluate the safety, tolerability, maximum tolerated dose (MTD)/recommended phase II dose (RP2D), and pharmacokinetics (PK) of TAK-164, in adult patients with GCC-positive GI malignancies.

## Materials and methods

### Patients

Eligible patients were aged ≥ 18 years, with histologically or cytologically confirmed GCC-positive (*H*-score ≥ 10 as indicated by IHC) GI cancers for whom standard treatment was no longer effective, or not available. Eligible GI malignancies included, but were not limited to: metastatic colorectal carcinoma, gastric carcinoma, esophageal carcinoma, small intestine cancer, and pancreatic cancer. Patients were required to have an Eastern Cooperative Oncology Group performance status of 0–1, a life expectancy of at least 12 weeks, and must have completed prior chemotherapy, biologic therapy, immunotherapy, or radiotherapy at least 4 weeks prior to enrollment. Additional inclusion criteria were: adequate bone marrow function (absolute neutrophil count [ANC] of ≥ 1.5 × 10^9^/L, platelet count ≥ 100 × 10^9^/L, and hemoglobin ≥ 9 g/dL), adequate hepatic function (total bilirubin ≤ 1.5 × upper limit of normal [ULN]; serum alanine aminotransferase [ALT], and aspartate aminotransferase [AST] ≤ 2.5 × ULN; serum albumin ≥ 3.0 g/dL) and adequate renal function defined by creatinine clearance ≥ 60 mL/min. Patients were excluded if they had: received anticancer chemotherapy or biologic therapy, or treatment with an experimental anticancer agent within 28 days, or received a diagnosis or treatment for another malignancy within 2 years, before administration of the first TAK-164 dose; a chronic or active infection requiring systemic therapy, as well as a history of symptomatic viral infection which has not been fully cured; a symptomatic central nervous system malignancy or metastasis or had been previously diagnosed with another malignancy, and any evidence of residual disease. Patients with non-melanoma skin cancer or carcinoma in situ of any type were not excluded if they had undergone complete resection. Additional exclusion criteria were: history of congestive failure with New York Heart Association class > 2, unstable angina (within 3 months of study enrollment), recent myocardial infarction (within 6 months of study enrollment), transient ischemic attacks, stroke, arterial or venous vascular disease, or clinically significant symptomatic arrhythmia despite anti-arrhythmic therapy; corrected QT by Fridericia method interval > 470 ms; and use of strong cytochrome P450 3A inhibitors, inducers, or modulators of P-glycoprotein or breast cancer resistance protein, within 1 week before the first TAK-164 dose.

### Study design

This was an open-label, multicenter, first in-human, phase I dose-escalation study of TAK-164. Patients received TAK-164 as a single intravenous infusion with a duration of up to 2 h, on day 1 of each 21-day cycle or every 3 weeks, until disease progression, unacceptable toxicity, or withdrawal from the study. Doses of 0.004 mg/kg, 0.008 mg/kg, 0.016 mg/kg, 0.032 mg/kg, 0.064 mg/kg, 0.12 mg/kg, 0.16 mg/kg, 0.19 mg/kg, 0.25 mg/kg, and 0.32 mg/kg were planned. To guide dose escalation, a method based on a Bayesian model of modified toxicity probability interval (mTPI) was used. Dose-limiting toxicities (DLTs) occurring during the first cycle of treatment (cycle 1) at a given dose level were evaluated to determine dose escalation. Safety and tolerability data beyond cycle 1 were used to determine the RP2D. DLTs were defined as: grade 4 neutropenia (ANC < 500 cells/mm^3^); febrile neutropenia as characterized by an ANC < 1000/mm^3^ and a single temperature of > 38.3 °C or a sustained temperature of ≥ 38 °C for more than 1 h; grade 4 thrombocytopenia (platelets < 25,000/mm^3^); grade ≥ 3 thrombocytopenia with clinically meaningful bleeding at any time; grade ≥ 3 nausea and/or emesis that occurred despite the use of optimal anti-emetic prophylaxis; grade ≥ 3 diarrhea that occurred despite optimal supportive care measures; any other grade ≥ 3 non-hematologic toxicity (except for brief [< 1 week] grade 3 fatigue); inability to start the next cycle of therapy due to treatment delay of more than 2 weeks because of a lack of adequate recovery of TAK-164-related hematological or non-hematologic toxicities; other TAK-164-related non-hematologic toxicities grade ≥ 2 that, in the opinion of the investigator, required discontinuation of TAK-164.

The primary objective was to evaluate the safety of TAK-164 and determine the MTD/RP2D. Secondary objectives were to characterize TAK-164 PK and immunogenicity, and to assess efficacy as measured by ORR. Exploratory objectives included assessment of the pharmacodynamic effect of TAK-164 in tumor biopsies.

### Assessments

Treatment-emergent adverse events (TEAEs) were assessed after administration of the first dose of TAK-164 and through 30 days after the last dose of TAK-164, were graded according to the National Cancer Institute Common Terminology Criteria for Adverse Events (NCI CTCAE) version 5 and were tabulated according to the Medical Dictionary for Regulatory Activities (MedDRA). Response was assessed based on the modified Response Evaluation Criteria in Solid Tumors (RECIST) version 1.1, at screening, every 2 cycles, and at end of treatment (defined as 30 days after the last TAK-164 dose, or prior to the start of subsequent antineoplastic therapy). The proportion of patients with positive anti-drug antibodies (ADA; transient and persistent, titer and specificity) was assessed in patients with baseline and at least one post-baseline immunogenicity assessment. Serial blood samples to determine the plasma concentration and PK of TAK-164 were taken pre-dose and post-dose on day 1 and on days 2, 3, 4, 8, and 15 of cycles 1 and 2, as well as pre- and post-dose on day 1 of cycle 3 onwards.

Archived tumor specimens and pre-treatment biopsies were used for analysis using a validated, semi-quantitative GCC IHC assay to confirm the presence of GCC protein expression, which was characterized by an H-score. Samples with apical H-scores ≥ 10 were considered “GCC-positive”. Target engagement of TAK-164 was assessed by measuring changes in expression of the target marker γH2AX using a validated IHC assay (NeoGenomics) and image analysis solution (Indica Labs) in paired pre- and post-treatment biopsies, obtained at screening and on day 8 of cycle 2.

### Sample size and statistical analyses

Approximately, 25 patients were planned to be enrolled. The mTPI dosing schema was planned to maximize patients treated at or near the MTD. Any excessive toxicity, defined as the probability of the DLT level exceeding 25% being greater than 95%, would prevent the escalation to next or higher dose levels and would lead to early termination of the study.

Statistical analyses were primarily descriptive and graphical in nature with no formal hypothesis testing. The safety population included all patients who received at least one dose of TAK-164. The response-evaluable population included all patients with measurable disease who received ≥ 1 dose of TAK-164 and had ≥ 1 post-baseline response assessment. The PK-evaluable population was defined as all patients with sufficient dosing and PK data to reliably estimate PK parameters.

## Results

### Patient disposition and baseline characteristics

From a total of 300 patients who were pre-screened for GCC expression, 31 were enrolled at five sites in the USA. Among all pre-screened patients, 218 were GCC-positive, 62 were GCC-negative, and 20 were undetermined. Among the 218 GCC-positive patients, 147 had a high baseline apical *H*-score (≥ 150). Baseline apical GCC expression varied among the 31 GCC-positive patients who met eligibility criteria for enrollment (Supplementary Fig. S1); 22 had a high apical *H*-score, and 9 had a low apical *H*-score (< 150).

Patient baseline demographics and disease characteristics among the 31 patients included in the study are summarized in Table 1. The median age was 58 years (range 32–72) and the majority of patients had colorectal carcinoma (80.6%) and stage IV disease (96.8%). The median time since diagnosis was 4 years (range 1–9) and the overall median prior lines of therapy was four (range 2–9). All patients discontinued the study drug, most frequently due to disease progression (54.8%) followed by withdrawal and symptomatic deterioration (both 19.4%).

**Table 1:** Patient baseline demographics and disease characteristics

	TAK-164 dose, mg/kg										
	0.004 *n* = 1	0.008 *n* = 1	0.016 *n* = 1	0.032 *n* = 5	0.064 *n* = 7	0.12 *n* = 7	0.16 *n* = 2	0.19 *n* = 3	0.25 *n* = 3	0.32 *n* = 1	Total *N* = 31
Median age, years (range)	72	52	59	51 (47–67)	61 (41–64)	49 (32–64)	53 (43–63)	64 (63–69)	47 (45–52)	39	58 (32–72)
Female, *n* (%)	1 (100)	1 (100)	1 (100)	4 (80.0)	4 (57.1)	4 (57.1)	1 (50.0)	1 (33.3)	0	1 (100)	18 (58.1)
Tumor type, *n* (%)											
Colorectal carcinoma	1 (100)	1 (100)	1 (100)	3 (60.0)	4 (57.1)	6 (85.7)	2 (100)	3 (100)	3 (100)	1 (100)	25 (80.6)
Esophageal carcinoma	0	0	0	1 (20.0)	1 (14.3)	0	0	0	0	0	2 (6.5)
Gallbladder carcinoma	0	0	0	1 (20.0)	0	0	0	0	0	0	1 (3.2)
Mucinous adenocarcinoma of unknown primary	0	0	0	0	0	1 (14.3)	0	0	0	0	1 (3.2)
Peritoneum	0	0	0	0	1 (14.3)	0	0	0	0	0	1 (3.2)
Omentum, ovarian	0	0	0	0	1 (14.3)	0	0	0	0	0	1 (3.2)
MSI status, *n* (%)											
MSI—High	1 (100)	0	1 (100)	0	0	0	0	1 (33.3)	2 (66.7)	0	5 (16.1)
MSS	0	0	0	3 (60.0)	4 (57.1)	5 (71.4)	1 (50.0)	2 (66.7)	1 (33.3)	1 (100)	17 (54.8)
Unknown	0	1 (100)	0	2 (40.0)	3 (42.9)	2 (28.6)	1 (50.0)	0	0	0	9 (29.0)
Disease stage at study entry											
III	0	0	0	0	0	1 (14.3)	0	0	0	0	1 (3.2)
IV	1 (100)	1 (100)	1 (100)	5 (100)	7 (100)	6 (85.7)	2 (100)	3 (100)	3 (100)	1 (100)	30 (96.8)
Median time since initial diagnosis, years (range)	3.0	8.0	7.0	2.5 (1–3)	4.0 (2–7)	4.0 (3–9)	4.5 (4–5)	3.0 (2–6)	5.0 (2–7)	4.0	4.0 (1–9)
Number of prior lines of therapy, *n* (%)											
2	0	0	0	1 (20.0)	0	0	0	0	0	0	1 (3.2)
3	0	0	0	2 (40.0)	1 (14.3)	1 (14.3)	0	0	1 (33.3)	0	5 (16.1)
4	0	0	0	1 (20.0)	6 (85.7)	2 (28.6)	0	1 (33.3)	0	0	10 (32.3)
5	0	1 (100)	1 (100)	0	0	1 (14.3)	0	1 (33.3)	0	0	4 (12.9)
6+	1 (100)	0	0	1 (20.0)	0	3 (42.9)	2 (100)	1 (33.3)	2 (66.7)	1 (100)	11 (35.5)
Median number of prior lines of therapy (range)	6	5	5	3 (2–7)	4 (3–4)	5 (3–9)	6.5 (6–7)	5 (4–7)	6 (3–7)	6	4 (2–9)
ECOG performance status, *n* (%)											
0	1 (100)	0	1 (100)	2 (40.0)	4 (57.1)	4 (57.1)	0	0	1 (33.3)	1 (100)	14 (45.2)
1	0	1 (100)	0	2 (40.0)	3 (42.9)	3 (42.9)	2 (100)	3 (100)	2 (66.7)	0	16 (51.6)
2	0	0	0	1 (20.0)	0	0	0	0	0	0	1 (3.2)
KRAS, *n* (%)											
Mutant	0	0	1 (100)	3 (60.0)	3 (42.9)	4 (57.1)	1 (50.0)	1 (33.3)	2 (66.7)	1 (100)	16 (51.6)
Wild type	1 (100)	1 (100)	0	2 (40.0)	3 (42.9)	2 (28.6)	1 (50.0)	2 (66.7)	1 (33.3)	0	13 (41.9)
Unknown	0	0	0	0	1 (14.3)	1 (14.3)	0	0	0	0	2 (6.5)
Missing	0	0	0	0	0	0	0	0	0	0	0

*ECOG* Eastern Cooperative Oncology Group, *KRAS* Kirsten rat sarcoma viral oncogene homolog, *MSI* microsatellite instability, *MSS* microsatellite stable

### Dose escalation and safety analysis

Patients received TAK-164 at the following dose levels: 0.004 mg/kg (*n* = 1), 0.008 mg/kg (*n* = 1), 0.016 mg/kg (*n* = 1), 0.032 mg/kg (*n* = 5), 0.064 mg/kg (*n* = 7), 0.12 mg/kg (*n* = 7), 0.16 mg/kg (*n* = 2), 0.19 mg/kg (*n* = 3), 0.25 mg/kg (*n* = 3), and 0.32 mg/kg (*n* = 1). Patients across all dose cohorts received a median number of 2 (range 1–8) treatment cycles, with 71% of patients receiving at least 2 cycles. The median treatment duration was 6 weeks (range 3.0–27.1), with a median relative dose intensity of 100% (range 60–102.4). No DLTs were reported during cycle 1, allowing dose escalation to proceed to 0.32 mg/kg. Three patients reported TEAEs during cycle 2 dosing considered to be DLT signals by the investigators. One of the 3 patients receiving TAK-164 at 0.19 mg/kg experienced grade 3 pyrexia and a grade 5 cholestatic hepatic failure which occurred 50 days after the last dose of TAK-164 and was considered related to study treatment; this patient had a history of liver resection and cholecystectomy due to hepatic metastases and stents which were found to be occluded on endoscopic retrograde cholangio-pancreatography examination. Of the 3 patients receiving 0.25 mg/kg TAK-164, 1 experienced grade 3 nausea, grade 4 platelet count decrease, and grade 4 neutrophil count decrease, whilst another patient experienced grade 4 platelet count decrease and grade 4 hepatic failure (with elevations of ALT and AST from day 8 of cycle 1 with levels continuing to increase through to day 9 of cycle 2; and elevated bilirubin and alkaline phosphatase detected on day 8 of cycle 2). As this patient had no reported liver metastasis at baseline, the grade 4 hepatic failure was considered related to study treatment; the hepatic failure was not resolved and the drug was withdrawn. As a result of the hepatic toxicity events reported, the maximum TAK-164 dose was capped at 0.19 mg/kg and additional patients were enrolled at at 0.032 and 0.064 mg/kg, and subsequently at 0.12 and 0.16 mg/kg to determine the RP2D. Based on a holistic assessment of all available data the RP2D was determined as 0.064 mg/kg. Following the completion of dose escalation, the study was terminated because of concerns about hepatic toxicity and insufficient anti-tumor efficacy.

The overall safety profile of TAK-164 is shown according to dose level received in Table 2, and the most frequently reported TAK-164-related all-grade and grade ≥ 3 TEAEs are shown in Table 3. As hepatic toxicity was identified as a safety signal during dose escalation, hepatic TEAEs are summarized in Supplementary Table S1. All patients reported at least one TEAE, 77.4% of patients experienced at least one TEAE related to TAK-164, and 32.3% of patients experienced at least one TAK-164-related grade ≥ 3 TEAE. The most common TEAEs related to TAK-164 were platelet count decreased (58.1%), fatigue (38.7%), anemia (32.3%), blood alkaline phosphatase increase (25.8%), AST increased, nausea, and vomiting (all 22.6%). The most frequently reported grade ≥ 3 TEAEs related to TAK-164 were platelet count decreased (12.9%), ALT increased, AST increase, fatigue, and anemia (all 9.7%). In total, 38.7% of patients experienced serious TEAEs and 16.1% reported serious TEAEs related to TAK-164, which included nausea, vomiting, pyrexia, hepatic failure, acute kidney injury, ALT increased, blood bilirubin increased, and platelet count decreased (each *n* = 1). Overall, 3 patients discontinued treatment as a result of TEAEs, all of which were related to TAK-164. One patient reported ALT increased, another patient reported AST increased and hyperbilirubinemia, and a third patient reported hepatic failure and platelet count decreased. None of the 31 patients died during the study, i.e., between the first dose of study through 30 days after the last dose of study drug. Two patients died more than 30 days after receiving the last TAK-164 dose. One patient died 57 days after the last dose due to disease complications. The other patient died 50 days after the last dose, as a result of grade 5 cholestatic hepatic failure, which was considered related to TAK-164. Only 1 patient was positive for ADA at baseline and one of the collected post-baseline samples, with a titer lower than the minimum required dilution of 81, demonstrating that observed toxicity was not due to ADA.

**Table 2:** Overall summary of TEAEs across all TAK-164 doses (safety population)

TAK-164 dose, mg/kg											
*n* (%)	0.004 *n* = 1	0.008 *n* = 1	0.016 *n* = 1	0.032 *n* = 5	0.064 *n* = 7	0.12 *n* = 7	0.16 *n* = 2	0.19 *n* = 3	0.25 *n* = 3	0.32 *n* = 1	Total *N* = 31
TEAEs	1 (100)	1 (100)	1 (100)	5 (100)	7 (100)	7 (100)	2 (100)	3 (100)	3 (100)	1 (100)	31 (100)
Related to TAK-164	1 (100)	0	1 (100)	5 (100)	6 (85.7)	5 (71.4)	1 (50.0)	2 (66.7)	2 (66.7)	1 (100)	24 (77.4)
Grade ≥ 3 TEAEs	0	1 (100)	1 (100)	3 (60.0)	1 (14.3)	5 (71.4)	1 (50.0)	3 (100)	3 (100)	0	18 (58.1)
Related to TAK-164	0	0	1 (100)	1 (20.0)	1 (14.3)	3 (42.9)	1 (50.0)	1 (33.3)	2 (66.7)	0	10 (32.3)
Leading to discontinuation	0	0	0	0	0	2 (28.6)	0	0	1 (33.3)	0	3 (9.7)
Leading to dose reduction	0	0	0	0	1 (14.3)	1 (14.3)	0	0	0	0	2 (6.5)
Leading to dose delay	0	0	0	2 (40.0)	0	1 (14.3)	0	1 (33.3)	1 (33.3)	0	5 (16.1)
Serious TEAEs	0	1 (100)	0	3 (60.0)	0	3 (42.9)	1 (50.0)	2 (66.7)	2 (66.7)	0	12 (38.7)
Related to TAK-164	0	0	0	0	0	1 (14.3)	1 (50.0)	1 (33.3)	2 (66.7)	0	5 (16.1)

*TEAEs* treatment-emergent adverse events

**Table 3:** Any-grade and grade ≥ 3 TEAEs related to TAK-164 (safety population)

Preferred term, *n* (%)	All-grade *N* = (31)	Grade ≥ 3 *N* = (31)
Platelet count decrease	18 (58.1)	4 (12.9)
Fatigue	12 (38.7)	3 (9.7)
Anemia	10 (32.3)	3 (9.7)
Blood alkaline phosphatase increased	8 (25.8)	0
AST increased	7 (22.6)	3 (9.7)
Nausea	7 (22.6)	1 (3.2)
Vomiting	7 (22.6)	1 (3.2)
Blood bilirubin increased	4 (12.9)	2 (6.5)
ALT increased	3 (9.7)	3 (9.7)
Lymphocyte count decreased	3 (9.7)	0
Neutrophil count decreased	3 (9.7)	1 (3.2)
Constipation	3 (9.7)	0
Hepatic failure	2 (6.5)ª	2 (6.5)ª
INR increase	2 (6.5)	1 (3.2)
WBC count decrease	2 (6.5)	1 (3.2)
Pyrexia	2 (6.5)	1 (3.2)
Stomatitis	2 (6.5)	0
Arthralgia	1 (3.2)	0
Taste disorder	1 (3.2)	0
Insomnia	1 (3.2)	0
Dyspnea	1 (3.2)	0
Rash	1 (3.2)	0
Rash maculopapular	1 (3.2)	0
Hyperbilirubinemia	1 (3.2)	1 (3.2)
Hypomagnesemia	1 (3.2)	0
Hyponatremia	1 (3.2)	0
Hypophosphatemia	1 (3.2)	1 (3.2)
Acute kidney injury	1 (3.2)	0
Proteinuria	1 (3.2)	0
Dermatitis bullous	1 (3.2)	0
Dermatitis acneiform	1 (3.2)	0
Gastro-esophageal reflux disease	1 (3.2)	0
Diarrhea	1 (3.2)	0
Colitis	1 (3.2)	0
Abdominal pain	1 (3.2)	0
Abdominal distension	1 (3.2)	0
Mucosal inflammation	1 (3.2)	0
Injection site bruising	1 (3.2)	0
Infusion site hemorrhage	1 (3.2)	0
Extravasation	1 (3.2)	0
Chills	1 (3.2)	0
Blood creatinine increased	1 (3.2)	0
Lipase increased	1 (3.2)	0

*ALT* alanine transaminase, *AST* aspartate aminotransferase, *INR* internationalized normal ratio, *TEAEs* treatment-emergent adverse events, *WBC* white blood cell

^a^Includes a fatal serious adverse event at 50 days post final dose of TAK-164

### Response assessment

Overall, 25 patients had received at least one dose of TAK-164 and had at least one post-baseline response assessment and so were evaluable for response. One patient with colon cancer and high baseline apical GCC *H*-score of 200 who received 5 cycles of TAK-164 at 0.008 mg/kg reported an unconfirmed partial response at cycle 4, giving an ORR of 4%. Eleven patients (44%) had a best overall response of stable disease (apical GCC H-score range: 20–280), giving a disease control rate of 48%. The apical GCC H-score among the 13 patients with progressive disease ranged from 12 to 290. Three patients who received 8 cycles of TAK-164 had colon cancer (*n* = 2) and esophageal cancer (*n* = 1).

### PK analysis

**Fig. 1:**
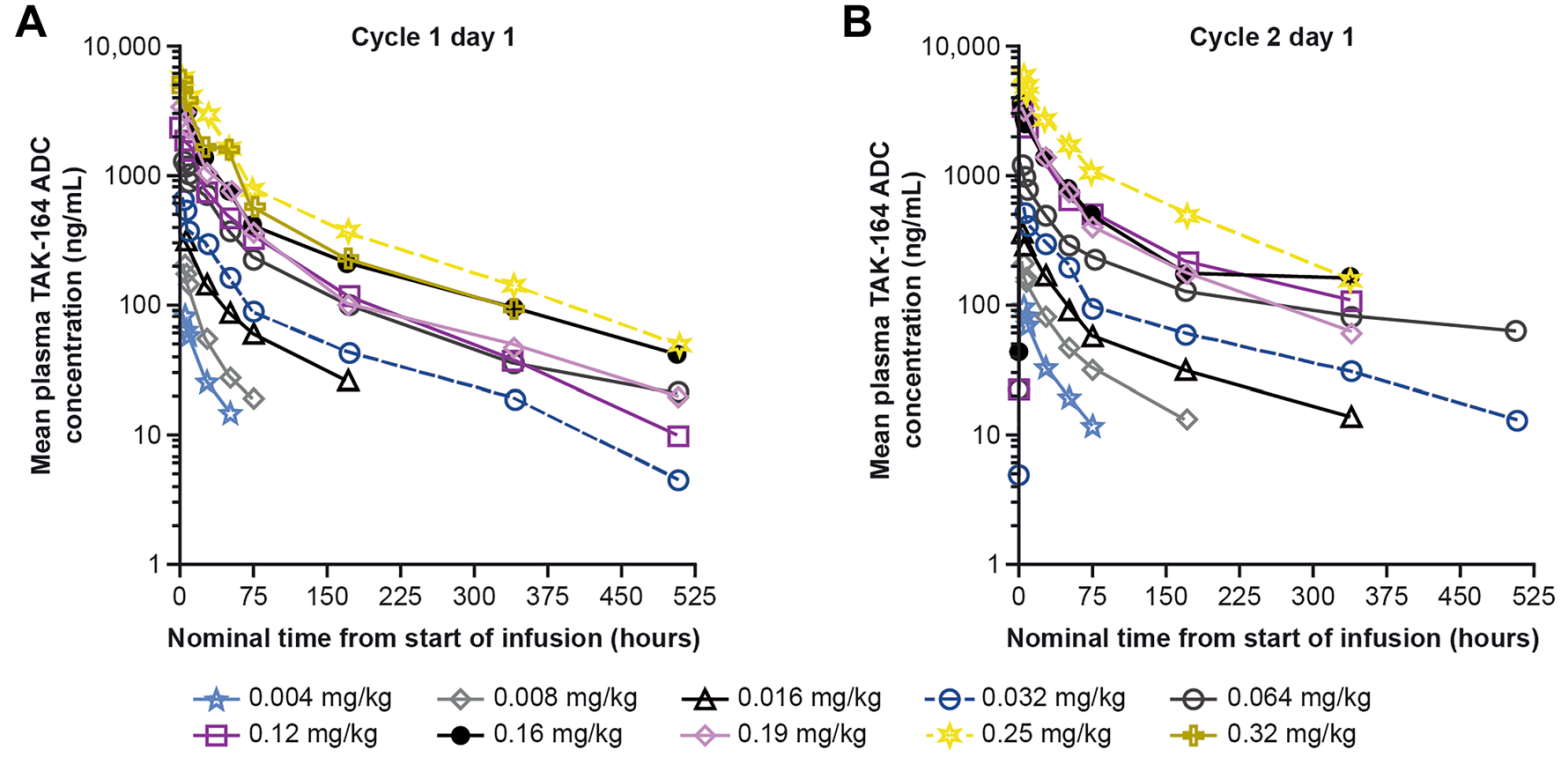
Plasma concentration–time profiles of TAK-164 at 0.004–0.32 mg/kg Q3W on Cycle 1 day 1 (**A**) and Cycle 2 day 1 (**B**). Semi-log plots grouped by dose and segregated by cycle. *Q3W* every 3 weeks

**Fig. 2:**
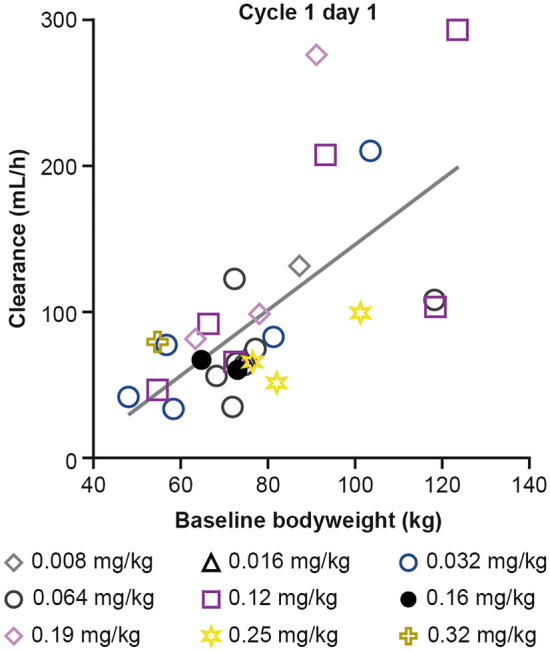
TAK-164 plasma clearance^a^ versus patient baseline bodyweight. ^a^Due to lack of sufficient data, clearance was not estimated for the patient who received the 0.004 mg/kg dose

TAK-164 concentration–time profiles were consistent with an intravenous infusion, with peak concentrations generally occurring at (or near) the time of end of the infusion (Fig. 1). The disposition phase appeared to be bi-phasic (Fig. 1) and there was an increase in TAK-164 exposure (as per the maximum plasma concentration [*C*_max_] and area under the serum concentration‒time curve from zero to infinity [AUC_0–inf_]) with increasing dose (Supplementary Fig. S2). Overall, there were no meaningful differences in the PK of TAK-164 during cycles 1 and 2 after two doses with administration every 3 weeks. Approximately, dose-dependent increases in *C*_max_ and AUC_0–inf_ were observed with increasing dose (0.032 mg/kg to 0.25 mg/kg; Supplementary Fig. S2). The volume of distribution was slightly larger than plasma volume (50 to 55 mL/kg) based on cycle 1 data and was similar between cycle 2 and cycle 1. The estimated mean terminal disposition half-life of TAK-164 was variable and ranged from 63.5 to 159 h. A relationship between clearance and body weight was observed (Fig. 2), suggesting that body weight-based dosing is appropriate for TAK-164.

### Target engagement assessment

**Fig. 3:**
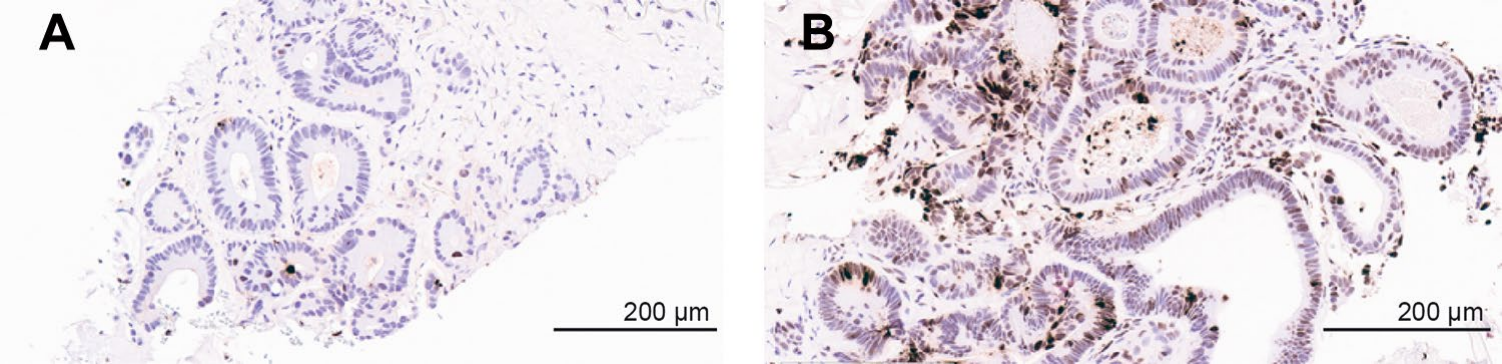
Biopsy imaging for γH2AX staining before baseline (**A**) and post-treatment (**B**) treatment with 0.19 mg/kg TAK-164

Paired biopsies from 2 patients were available to assess target engagement before and after treatment with TAK-164. Since the payload of TAK-164 is an alkylating agent, we utilized the DNA damage marker, γH2AX, as a surrogate marker of target engagement. Analysis of γH2AX staining from a patient with colon cancer receiving 0.19 mg/kg demonstrated target engagement with limited γH2AX staining present at baseline, and clear induction of γH2AX staining present following TAK-164 treatment (Fig. 3). Conversely, analysis of γH2AX staining from a patient with colon cancer receiving a lower dose of TAK-164 (0.064 mg/kg) did not demonstrate target engagement. Interpretation of this patient’s results was challenging because the pre-treatment biopsy had evidence of significant DNA damage (γH2AX staining) visible at baseline (Supplementary Figure S3).

## Discussion

ADCs are becoming increasingly prominent for the treatment of hematologic and solid tumor malignancies, with eleven ADCs approved globally, six of which, including the human epidermal growth factor receptor 2-directed ADC Enhertu, were approved since 2019 [17, 18]. The ADC TAK-164 consists of a human IgG1 mAb specifically targeting GCC, linked to a novel class of DNA alkylating agents termed IGNs, by a peptide linker [1]. In this first in-human, phase I study, a total of 31 patients with GCC-positive GI cancers were treated with TAK-164 across the dose range of 0.004–0.32 mg/kg every 3 weeks. No DLTs were observed in cycle 1 for all 31 patients. However, during extended safety and tolerability monitoring in cycle 2, significant hepatic toxicities were identified and considered related to study treatment: a grade 5 hepatic failure (50 days after the last dose) in 1 patient receiving 0.19 mg/kg, and a grade 4 hepatic failure (with elevations of ALT, AST, and bilirubin) in 1 patient dosed with 0.25 mg/kg. The sponsor hence determined hepatic toxicity as a potential risk, resulting in a dose cap at 0.19 mg/kg.

Overall, TAK-164 had a manageable safety profile with no hepatic toxicity observed up to the dose level of 0.064 mg/kg every 3 weeks. Across all dose levels being evaluated, almost a third of patients reported grade ≥ 3 TEAEs related to TAK-164, but only 3 patients discontinued treatment due to TAK-164-related TEAEs, whilst 2 patients required a dose reduction and 5 patients had to delay their TAK-164 dose. The most frequently reported all-grade TEAEs related to TAK-164 included GI toxicities (nausea and vomiting, both occurring in 22.6% of patients). These are commonly reported in patients with GI cancers and were consistent with the TAK-164 mode of action, and with the TEAEs observed in TAK-264 clinical trials [13, 15]. Hematologic toxicity was also frequently observed, with TAK-164-related platelet count decrease and anemia occurring in 58.1% and 32.3% of patients, respectively. Hematologic TEAEs were similarly reported with TAK-264 and are likely a result of free payload, since GCC expression has not been reported in bone marrow cells [14].

Serious hepatic toxicities were reported at dose levels above the RP2D; these were unexpected since GCC expression has not been observed in hepatocytes, with the exception of a single study in rats where GCC was shown to be expressed in regenerating hepatocytes [19]; moreover, hepatic toxicity was not observed in phase I TAK-264 trials [13, 15]. A possible reason for the reported toxicity could be the expression of GCC in liver metastases derived from primary colorectal cancer in certain patients, as consistent GCC expression has been demonstrated in primary and matched metastatic lesions of colorectal cancer tissues from the same patients [7]. Alternatively, as cleavable linkers have been previously recognized as unstable [20], the hepatic toxicity observed in this study may have been as a result of off-target effects from the cytotoxic payload being released outside of the target tissue. The alanine–alanine dipeptide linker in TAK-164 is designed to be cleaved intracellularly once entering target cells [16], reducing the risk of off-target payload release; however, it is possible that the peptide linker is not as stable in systemic circulation as expected. Alternatively, toxicity may occur as a result of hepatic uptake and local/systemic release of the payload, on top of the inability to excrete the payload due to its own properties and mechanism of action. Further pharmacokinetics analyses of free payload are needed to support these hypotheses.

Based on the promising preclinical data in a GCC-expressing human HEK293 cell line and a mouse xenograft model [1], it was anticipated that the TAK-164 payload might result in improved clinical outcomes versus the limited clinical activity observed with TAK-264 [14, 15]. However, this was not observed clinically, with only 1 of 25 response-evaluable patients achieving an unconfirmed partial response, and 11 patients achieving stable disease. Given that the patients were heavily pretreated and were diagnosed with advanced disease, there was a high threshold for achieving meaningful clinical responses in this patient population. The RP2D of 0.064 mg/kg, was considered to be too low to provide significant clinical benefit to patients, and higher doses were too toxic. Therefore, a decision was made to terminate the study.

Collection of biopsies throughout the trial, particularly post-treatment, was challenging; only two pairs of pre-treatment and on-treatment biopsies were collected at the 0.064 mg/kg and the 0.19 mg/kg dose levels. The patient samples from the 0.19 mg/kg dose level showed robust target engagement consistent with those seen in preclinical models. At the 0.064 mg/kg dose level, the baseline sample had high levels of γH2AX, most likely due to smoking status, highlighting a limitation of the DNA damage marker as it captures any instance of DNA damage and not necessarily TAK-164-induced DNA damage. The varied GCC expression across the patient cohort was in line with previously published GCC expression data in colorectal, gastric, and esophageal carcinomas [7].

In conclusion, intravenously administered TAK-164 up to 0.064 mg/kg had a manageable safety profile in patients with various GCC-positive GI cancers. Given the hepatic toxicity signal at higher TAK-164 doses and insufficient clinical benefit observed, further evaluation of single-agent TAK-164 is not warranted in this setting. ADCs are a rapidly evolving class of drugs; considering the existing preclinical evidence for the anti-tumor effect of targeting GCC, future development and investigation of novel anti-GCC ADCs, as single agents or in combination with existing therapy regimens, may lead to clinically effective anticancer treatments.

## Supplementary Information

Below is the link to the electronic supplementary material.

Supplementary file1 (DOCX 1599 KB)

## Data Availability

The datasets, including the redacted study protocol, redacted statistical analysis plan, and individual participants’ data supporting the results reported in this article, will be made available within 3 months from initial request, to researchers who provide a methodologically sound proposal. The data will be provided after its de-identification, in compliance with applicable privacy laws, data protection and requirements for consent and anonymization.
