# Correction to: A phase I, first-in-human study of TAK-164, an antibody–drug conjugate, in patients with advanced gastrointestinal cancers expressing guanylyl cyclase C

**DOI:** 10.1007/s00280-023-04522-x

**Published:** 2023-03-26

**Authors:** Richard Kim, Alexis D. Leal, Aparna Parikh, David P. Ryan, Shining Wang, Brittany Bahamon, Neeraj Gupta, Aaron Moss, Joanna Pye, Harry Miao, Haig Inguilizian, James M. Cleary

**Affiliations:** 1 Department of Gastroenterology Oncology, Moffitt Cancer Center, Vincent A. Stabile Research Building, 12902 USF Magnolia Drive, Tampa, FL 33612 USA; 2 Division of Medical Oncology, University of Colorado School of Medicine, Aurora, CO USA; 3 Division of Hematology and Oncology, Massachusetts General Hospital Cancer Center, Harvard Medical School, Boston, MA USA; 4 Oncology Clinical Science, Takeda Development Center Americas, Inc. (TDCA), Lexington, MA USA; 5 Translational Medicine, Takeda Development Center Americas, Inc. (TDCA), Lexington, MA USA; 6 Quantitative Clinical Pharmacology, Takeda Development Center Americas, Inc. (TDCA), Lexington, MA USA; 7 Pharmacology/Toxicology, Audentes Therapeutics, Inc., San Francisco, CA USA; 8 Oncology Statistics, Takeda Development Center Americas, Inc. (TDCA), Lexington, MA USA; 9 Clinical Development, Takeda Development Center Americas, Inc. (TDCA), Lexington, MA USA; 10 Global Patient Safety and Evaluation, Takeda Development Center Americas, Inc. (TDCA), Lexington, MA USA; 11 Department of Medical Oncology, Dana-Farber Cancer Institute, Harvard Medical School, Boston, MA USA

**Correction to: Cancer Chemotherapy and Pharmacology** 10.1007/s00280-023-04507-w

The article “A phase I, first-in-human study of TAK-164, an antibody–drug conjugate, in patients with advanced gastrointestinal cancers expressing guanylyl cyclase C”, written by Richard Kim · Alexis D. Leal · Aparna Parikh · David P. Ryan · Shining Wang · Brittany Bahamon ·Neeraj Gupta · Aaron Moss · Joanna Pye · Harry Miao · Haig Inguilizian and James M. Cleary was originally published Online First without Open Access. With the author(s)' decision to opt for Open Choice the copyright of the article changed on 6th March 2023 to © The Author(s) 2023 and the article is forthwith distributed under the terms of the Creative Commons Attribution 4.0 International License (https://creativecommons.org/licenses/by/4.0/), which permits use, sharing, adaptation, distribution and reproduction in any medium or format, as long as you give appropriate credit to the original author(s) and the source, provide a link to the Creative Commons licence, and indicate if changes were made.

The original article has been corrected.

