# Supplementary material for: A phase I, first-in-human study of TAK-164, an antibody–drug conjugate, in patients with advanced gastrointestinal cancers expressing guanylyl cyclase C
Source: Cancer Chemother Pharmacol. 2023 Feb 4;91(4):291–300. doi: 10.1007/s00280-023-04507-w (PMC10068631; doi:10.1007/s00280-023-04507-w)

# Supplementary Material

# Journal: *Cancer Chemotherapy and Pharmacology*

# A phase I, first-in-human study of TAK-164, an antibody-drug conjugate, in patients with advanced gastrointestinal cancers expressing guanylyl cyclase C

# Richard Kim^1^ ∙ Alexis D. Leal^2^ ∙ Aparna Parikh^3^ ∙ David P. Ryan^3^ ∙ Shining Wang^4^ ∙ Brittany Bahamon^5^ ∙ Neeraj Gupta^6^ · Aaron Moss^7^ ∙ Joanna Pye^8^ · Harry Miao^9^ ∙ Haig Inguilizian^10^ ∙ James M. Cleary^11^

# ^1^Department of Gastroenterology Oncology, Moffitt Cancer Center, Tampa, FL, USA ^2^Division of Medical Oncology, University of Colorado School of Medicine, Aurora, CO, USA ^3^Division of Hematology and Oncology, Massachusetts General Hospital Cancer Center, Harvard Medical School, Boston, MA, USA ^4^Oncology Clinical Science, Takeda Development Center Americas, Inc. (TDCA), Lexington, MA, USA ^5^Translational Medicine, Takeda Development Center Americas, Inc. (TDCA), Lexington, MA, USA ^6^Quantitative Clinical Pharmacology, Takeda Development Center Americas, Inc. (TDCA), Lexington, MA, USA ^7^Pharmacology/Toxicology, Audentes Therapeutics, Inc., San Francisco, CA, USA ^8^Oncology Statistics, Takeda Development Center Americas, Inc. (TDCA), Lexington, MA, USA ^9^Clinical Development, Takeda Development Center Americas, Inc. (TDCA), Lexington, MA, USA ^10^Global Patient Safety and Evaluation, Takeda Development Center Americas, Inc. (TDCA), Lexington, MA, USA ^11^Department of Medical Oncology, Dana-Farber Cancer Institute, Harvard Medical School, Boston, MA, USA

# Corresponding Author Richard Kim, Department of Gastroenterology Oncology, Moffitt Cancer Center, Vincent A. Stabile Research Building, 12902 USF Magnolia Drive, Tampa, FL 33612, United States. Phone: +1 813-745-4673. E-mail: [richard.kim@moffitt.org](mailto:richard.kim@moffitt.org).

## Supplementary Table S1. Observed hepatic toxicity events related to TAK-164 (safety population, *N =* 31)

| ***n* (%)** | **TAK-164-related** | **TAK-164-related grade ≥ 3** | **TAK-164-related serious** |
| --- | --- | --- | --- |
| Aspartate aminotransferase increased | 7 (22.6) | 3 (9.7) | 0 |
| Blood bilirubin increasedª | 5 (16.1) | 3 (9.7) | 1 (3.2) |
| Alanine aminotransferase increased | 3 (9.7) | 3 (9.7) | 1 (3.2) |
| Hepatic failure | 2 (6.5)ᵇ | 2 (6.5)ª | 2 (6.5)ᵇ |

ªIncludes hyperbilirubinemia
ᵇIncludes a fatal serious adverse event at 50 days post final dose of TAK-164

## Supplementary Figure S1. GCC apical H-score distribution among patients enrolled in the study (*N =* 31) by TAK-164 dose received


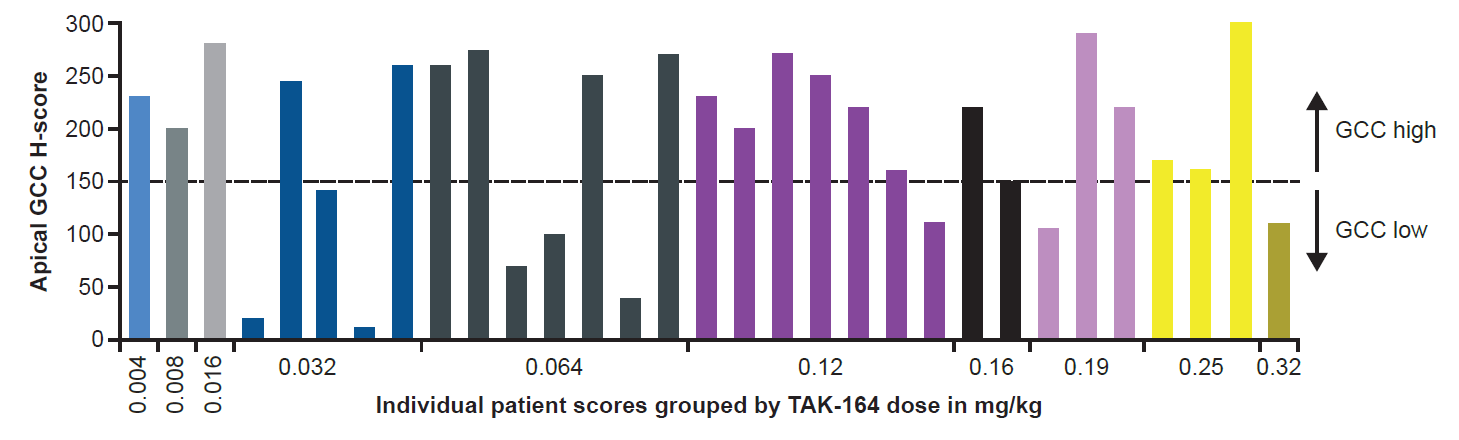
*GCC* guanylyl cyclase C

## Supplementary Figure S2. TAK-164 dose proportionality plots of C_max_ on Cycle 1 day 1 (A) and Cycle 2 day 1 (C) and AUC on Cycle 1 day 1 (B) and Cycle 2 day 1 (D), grouped by dose

##
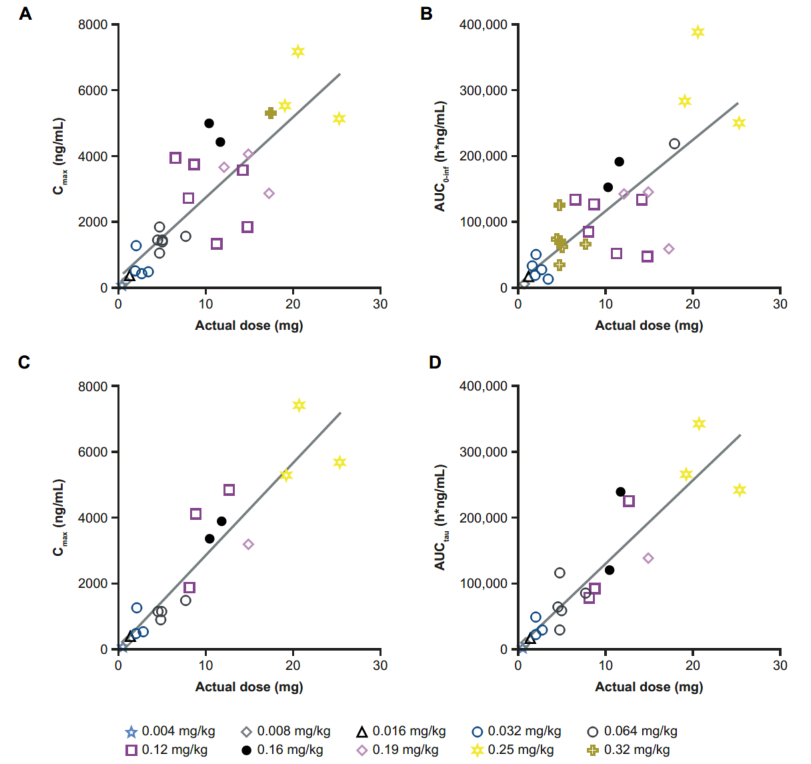
 *AUC* concentration-time curve, *AUC_0-inf_* area under the serum concentration-time curve from zero to infinity, *AUC_tau_* area under the serum concentration-time curve over the dosing interval, *C_max_* maximum plasma concentration

## Supplementary Figure S3. Biopsy imaging for γH2AX staining at baseline (A) and post-treatment (B) treatment with 0.064 mg/kg TAK-164


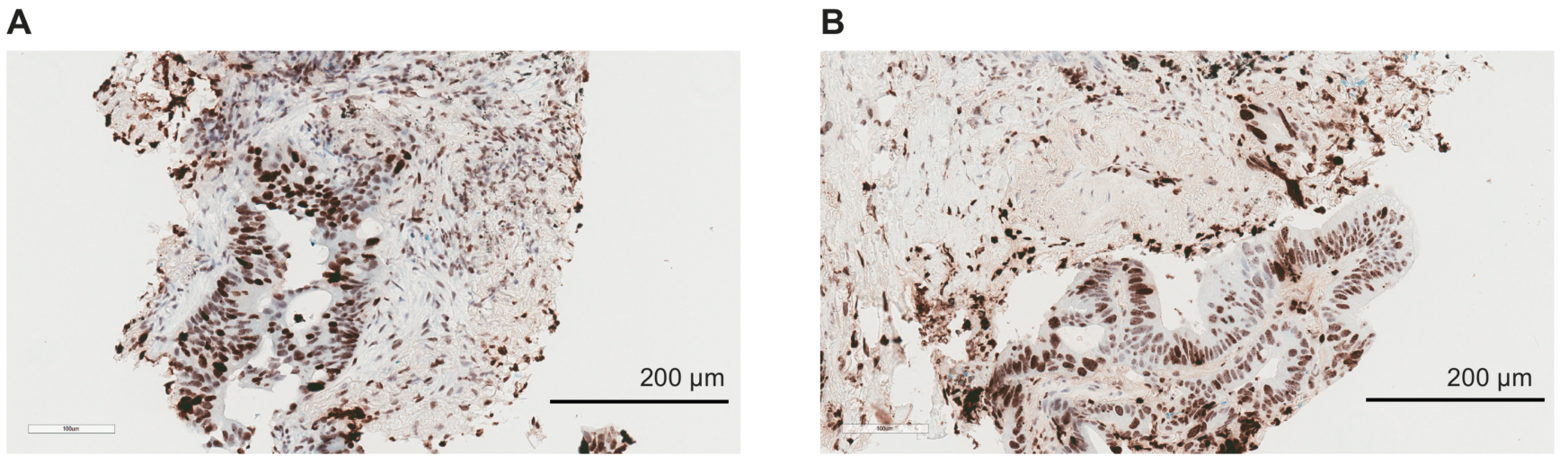

Supplement: Supplementary file 1 — Supplementary file1 (DOCX 1599 KB) [file 280_2023_4507_MOESM1_ESM.docx]
